# BAP1 cancer syndrome: malignant mesothelioma, uveal and cutaneous melanoma, and MBAITs

**DOI:** 10.1186/1479-5876-10-179

**Published:** 2012-08-30

**Authors:** Michele Carbone, Laura Korb Ferris, Francine Baumann, Andrea Napolitano, Christopher A Lum, Erin G Flores, Giovanni Gaudino, Amy Powers, Peter Bryant-Greenwood, Thomas Krausz, Elizabeth Hyjek, Rachael Tate, Joseph Friedberg, Tracey Weigel, Harvey I Pass, Haining Yang

**Affiliations:** 1 University of Hawai‘i Cancer Center, 677 Ala Moana Boulevard, Suite 901, Honolulu, 96813, HI, USA; 2 Pathology Department, University of Hawai‘i at Mānoa John. A. Burns School of Medicine, 651 Ilalo Street, MEB 401, Honolulu, 96813, HI, USA; 3 Department of Molecular Biosciences and Bioengineering, University of Hawai‘i at Mānoa, 1955 East–west Road, Agricultural Science 218, Honolulu, 96822, HI, USA; 4 Department of Pathology, The Queen’s Medical Center, 1301 Punchbowl Street, Honolulu, HI, 96813, USA; 5 Molecular Diagnostics and Biorepository, The Queen’s Medical Center, 1301 Punchbowl Street, Honolulu, 96813, HI, USA; 6 Department of Pathology, University of Pittsburgh, Presby South Tower Suite, 3880 200 Lothrop Street, Pittsburgh, 15213, PA, USA; 7 The University of Chicago Medicine, 5841 S. Maryland Avenue, Chicago, IL, 60637, USA; 8 Department of Pathology, The University of Chicago, 5841 S. Maryland Avenue, Chicago, TW-055, MC, IL, 60637, USA; 9 Private Practice Physician, 1270 Attakapas Drive, Opelousas, 70570, LA, USA; 10 Department of Surgery, Penn Presbyterian Medical Center, 266 Wright Saunders Building, 39th & Market Streets, Philadelphia, 19104, PA, USA; 11 Department of Surgery, University of Wisconsin School of Medicine and Public Health, 600 Highland Avenue, Clinical Science Center – H4, Madison, 53792-3284, WI, USA; 12 Department of Cardiothoracic Surgery, New York University, NYU Langone Medical Center, 160 East 34th Street, 8th Floor, New York, 10016, NY, USA

**Keywords:** BAP1, Mesothelioma, Melanoma, Cancer syndrome, MBAITs

## Abstract

**Background:**

BRCA1–associated protein 1 (*BAP1*) is a tumor suppressor gene located on chromosome 3p21. Germline *BAP1* mutations have been recently associated with an increased risk of malignant mesothelioma, atypical melanocytic tumors and other neoplasms. To answer the question if different germline *BAP1* mutations may predispose to a single syndrome with a wide phenotypic range or to distinct syndromes, we investigated the presence of melanocytic tumors in two unrelated families (L and W) with germline *BAP1* mutations and increased risk of malignant mesothelioma.

**Methods:**

Suspicious cutaneous lesions were clinically and pathologically characterized and compared to those present in other families carrying *BAP1* mutations. We then conducted a meta-analysis of all the studies reporting *BAP1*-mutated families to survey cancer risk related to the germline BAP1 mutation (means were compared using t-test and proportions were compared with Pearson χ^2^ test or two-tailed Fisher’s exact test).

**Results:**

Melanocytic tumors: of the five members of the L family studied, four (80%) carried a germline *BAP1* mutation (p.Gln684*) and also presented one or more atypical melanocytic tumors; of the seven members of W family studied, all carried a germline *BAP1* mutation (p.Pro147fs*48) and four of them (57%) presented one or more atypical melanocytic tumors, that we propose to call “melanocytic *BAP1*-mutated atypical intradermal tumors” (MBAITs). Meta-analysis: 118 individuals from seven unrelated families were selected and divided into a *BAP1*-mutated cohort and a *BAP1*-non-mutated cohort. Malignant mesothelioma, uveal melanoma, cutaneous melanoma, and MBAITs prevalence was significantly higher in the *BAP1*-mutated cohort (p ≤ 0.001).

**Conclusions:**

Germline *BAP1* mutations are associated with a novel cancer syndrome characterized by malignant mesothelioma, uveal melanoma, cutaneous melanoma and MBAITs, and possibly by other cancers. MBAITs provide physicians with a marker to identify individuals who may carry germline *BAP1* mutations and thus are at high risk of developing associated cancers.

## Background

Hereditary cancer syndromes are caused by mutations in genes conferring high relative risks of cancer among carriers [1]. The majority of the hereditary cancer syndromes are inherited in an autosomal dominant manner and the cancers occur early in life. Remarkably, benign mucocutaneous lesions (e.g. café-au-lait spots in neurofibromatosis type I, hamartomas in Cowden syndrome, and hyperpigmented macules in Peutz–Jeghers syndrome) are often the first clues that allow physicians to diagnose patients with these syndromes [2]. BAP1 (BRCA1–associated protein 1) is a member of the ubiquitin C-terminal hydrolase subfamily of deubiquitinating enzymes that catalyze the removal of ubiquitin from protein substrates [3], e.g. monoubiquitinated histone H2A [4]. Also, a multi-protein complex containing BAP1 is involved in regulation of cell transcription [5].

We recently identified two unrelated families (L and W) with germline *BAP1* mutations and increased risk of malignant mesothelioma (MM) and possibly other cancers [6]. In parallel with our study, Wiesner and colleagues reported germline *BAP1* mutations in two unrelated families with “atypical melanocytic tumors” and other cancers [7]. In a subsequent paper, Wiesner and colleagues called the same melanocytic lesions “atypical Spitz tumors” (ASTs), but noted that in contrast to ASTs these lesions had a different histology and were characterized by the presence of both BAP1 and BRAF mutations [8]. *BRAF* mutations are indeed common in melanocytic nevi (80%) and in cutaneous melanomas (CMs) (65%) but are rare in ASTs [8]. Other investigators independently confirmed the association of germline *BAP1* mutations and other cancers in additional families [9,10]. In two of these families, skin lesions morphologically, histologically, and molecularly similar to the previously reported “atypical melanocytic tumors” or “ASTs” were investigated and identified but instead called “nevoid melanoma-like melanocytic proliferations” (NEMMPs) [10].

To verify if germline *BAP1* mutations are associated with distinct syndromes or with a single syndrome exhibiting a wide phenotypic range, we first investigated the presence of melanocytic tumors in our two families and compared them to those published in the literature, and then conducted a pooled analysis of individuals from studies reporting *BAP1*-mutated families to compare cancer risk in the 63 mutated vs the 55 non-mutated individuals from those families.

We demonstrate that germline *BAP1* mutations are associated with a novel cancer syndrome characterized by MM, uveal melanoma (UVM), CM, MBAITs and possibly by other tumors.

## Methods

### Patients and family histories

We studied members of the L and W families. Written and informed consent was obtained from all participants in the study according to the guidelines set forth by the Institutional Review Board of the University of Hawai‘i. Family histories were collected through interviews, written questionnaires, and medical and/or pathological reports.

### Germline *BAP1* sequencing

Genomic DNA was extracted from whole blood and analyzed using bidirectional sequencing of the *BAP1* gene in our Hawaii Cancer Consortium CLIA/CAP certified laboratory.

### Clinical and pathological studies

*BAP1*-mutated family members were evaluated by a board certified dermatologist specialized in melanocytic lesions (L.K.F.). Biopsies from five members of L family (L-III-18, L-IV-4, L-IV-5, L-IV-13, and L-IV-15) and seven members of W family (W-III-4, W-IV-8, W-IV-9, W-IV-12, W-IV-13, W-IV-21, and W-IV-25) were collected. In total, 13 lesions were collected from the L family members and 11 from the W family members. A dermatoscopic analysis was conducted of all suspicious lesions after their excision.

Immunohistochemistry (IHC) was performed on paraffin tissue sections with a monoclonal antibody against BAP1 (1:200, C-4, Santa Cruz Biotechnology) using Leica-BOND III automated IHC and ISH system according to a modified manufacturer protocol using Bond Polymer Refine Detection kit (Leica Microsystems) following antigen retrieval Solution 1 (Leica Microsystems). The pathological evaluation of the tissue sections and the immune-staining were independently conducted at the University of Hawai‘i and at the University of Chicago with 100% diagnostic concordance.

Tumor tissue sections were microdissected with a laser capture microscope (Molecular Machine Industries) by a board certified dermatopathologist (C.A.L.). DNA was extracted and purified with a QIAamp DNA FFPE Tissue Kit (Qiagen) according to the manufacturer’s instructions. *BRAF* (exon15) mutations were detected by multiplex PCR amplification of tumor DNA, followed by automated fluorescent labeling, hybridization to a BioFilmChip Microarray, and signal detection using the AutoGenomics INFINITY Analyzer (AutoGenomics).

### Meta-analysis

To identify all reports of germline *BAP1*-mutated families, we searched the PubMed database using the search terms “germline” and “BAP1”. This search yielded 11 results. The inclusion criteria were: at least two generations and five members tested for germline *BAP1* mutations, one positive germline *BAP1* mutation found in at least one member of the family, and cancer status assessed for each of the tested members. Among the 11 results, four studies published between August 2011 and April 2012 satisfied the inclusion criteria, with a total of seven selected families [6,7,9,10]. Moreover, the findings of this current paper were included in the analysis. We asked the authors of the four selected papers for additional data: we collected gender, age of death or age of last follow up, germline *BAP1* mutation result, all diagnosed cancers, and age of each cancer diagnosis. These available data allowed us to avoid most of the problems arising with formal meta-analyses and to conduct a pooled analysis of the individual data. To assess if germline *BAP1* mutations were associated with an increased risk of cancer or MBAITs, we built two cohorts from these families: one cohort constituting 63 germline *BAP1*-mutated patients and the second cohort constituting 55 non-mutated patients. Both cohort numbers were higher than 30 cases, thus they were considered as following a normal distribution. Means were compared using the t-test if the Barlett’s test for equal variances was non-significant; otherwise means were compared by the Welch’s test. Proportions were compared with Pearson χ^2^ test, or two-tailed Fisher’s exact test when an expected number was fewer than five. Because of the very small numbers for each site of cancer, the odds ratio (OR) of only the overall cancer risk was calculated with its 95% confidence interval (CI). Statistical tests were performed with STATA (version 12.0) software.

## Results

**Figure 1:**
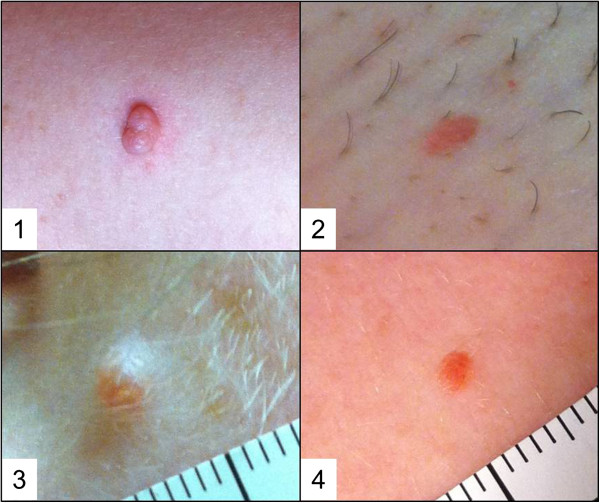
**Clinical presentations of MBAITs. **MBAITs have variable clinical presentations including pink polypoid papules (**1**, from shoulder of patient W-F1-IV-9), raised pink papules (**2**, from groin of L-F1-IV-15), and lightly pigmented papules (**3**, from cheek of L-F1-IV-4). These lesions were clinically difficult to distinguish from banal dermal nevi, as pictured in (**4**), which shows a clinically similar lesion also from the chest of the same patient pictured in (**2**).

We reviewed the published morphology and histology and some of the original tissue sections from the published melanocytic skin lesions [7-9]. In addition, we investigated for the presence of these melanocytic tumors in members of the *BAP1*-mutated families previously found to have developed MM and UVM [6]. These tumors were indistinguishable. Thus, pathologists have used different names to identify these pink to tan skin tumors of about 0.2-1.0 cm in diameter, which macroscopically resemble dermal nevi, but that histologically and at the molecular level do not fit any previous diagnostic nomenclature. In this paper we will use the term MBAITs (Melanocytic BAP1-mutated Atypical Intradermal Tumors) for all the described BAP1-mutated atypical melanocytic proliferations with similar histology and molecular characterization that were previously diagnosed using various terminologies (Figure 1).

Specifically, we analyzed suspicious cutaneous tumors from five members of L family and seven members of W family. Of the five members of L family, four (80%) presented one or more MBAITs. The age range of positively screened individuals was 36–64 years. One of them positive for MBAITs (L-III-18) had already been diagnosed with both MM and UVM.

Of the seven members of W family, four (57%) presented one or more MBAITs. The age range of the screened individuals was 26–61 years. One of them, positive for MBAITs (W-III-04), had already been diagnosed with MM. The individual W-IV-21, already diagnosed with MM, resulted negative for MBAITs screening, but reported a recently diagnosed breast carcinoma.

All patients communicated that all of the MBAITs looked and behaved as benign (present for a long time, not growing or changing).

**Figure 2:**
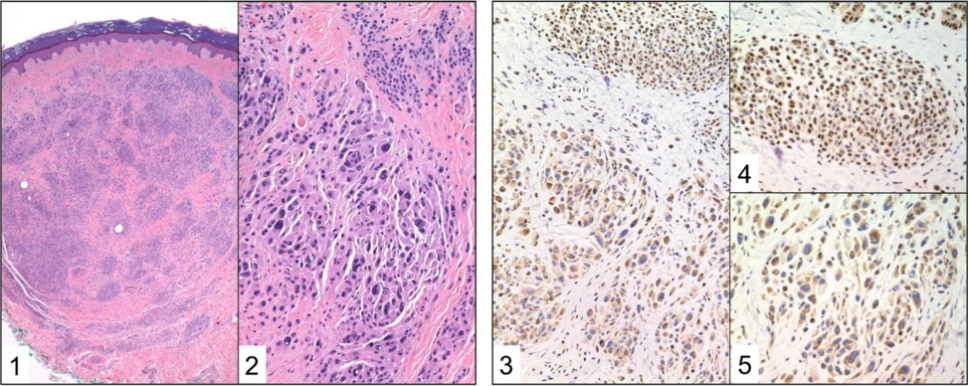
**Histology and immunohistochemistry of MBAITs.** Representative biopsy from individual W-III-04. The histologic examination (hematoxylin and eosin, H/E, of the nevi at the low power (H&E, 4X Original Magnification) magnification shows an intradermal melanocytic nevus with superficial nests and deeper single melanocytic units. This background melanocytic nevus shows maturation with depth. A second distinct, central, circumscribed population of melanocytes is identified at the mid-reticular dermis (**1**). This second population of melanocytes on higher power (H&E, 20X Original Magnification) shows variably sized, large epithelioid and spindled melanocytes. There is marked cytologic atypia comprised of pleomorphic, hyperchromatic nuclei (**2**). The immunohistochemistry for BAP1 demonstrates distinct staining between the background melanocytic nevus and the embedded clusters of atypical epithelioid melanocytes. Identified is strong nuclear positivity in the background melanocytic nevus and negative nuclear staining in the large epithelioid cells (**3**: BAP-1, 20X Original Magnification). On higher power, the background melanocytes show strong nuclear staining in this superficial nest (**4**: BAP-1, 40X Original Magnification). Large epithelioid cells with their pleomorphic nuclei demonstrate negative nuclear staining with variable cytoplasmic staining (**5**: BAP-1, 40X Original Magnification).

Histologically, MBAITs presented as intradermal lesions with large epithelioid and spindled melanocytes, with marked cytologic atypia and pleomorphic, hyperchromatic nuclei, and they were often associated with a nearby compound or intradermal nevus (Figure 2). The large epithelioid and spindle cells (i.e., the MBAITs cells) showed no mitotic activity, a finding confirmed by the negative Ki67 immunostaining (not shown). Kamino bodies were not identified. In the MBAITs cells –but not in the nearby smaller nevus cells- , IHC showed negative BAP1 nuclear staining with variable cytoplasmic staining, suggesting loss of heterozygosity (LOH) of the wild-type *BAP1* allele (Figure 2).

Of the five members of L family, four (80%) carried the germline *BAP1* mutation (p.Gln684*). The family member L-IV-5 did not have MBAITs and was not mutated in *BAP1*. All of the seven members of W family carried the germline *BAP1* mutations (p.Pro147fs*48).

*BRAF* genotyping was performed on all the nine diagnosed MBAITs and BRAF^V600E^ mutations were identified in all of them, confirming the extremely high prevalence of this mutation as originally reported by Wiesner and colleagues [7], and confirmed by Njauw and colleagues [10].

The characteristics of the pooled families are described in Table 1. Among the seven families, 118 patients were selected. The results of the meta-analysis are presented in Table 2. The *BAP1*-mutated cohort included 63 patients and the *BAP1*-non-mutated cohort 55 patients. The mean ages of follow up were comparable in both cohorts (53.2 years, 95%CI: 49.0-57.4 in the BAP1 mutated cohort, 51.0 years, 95%CI: 46.3-55.8 in the non-mutated cohort). The proportion of women was higher in the *BAP1*-mutated cohort compared to the non-mutated cohort (63.3% and 43.6% respectively, p = 0.034). At this time we do not have an explanation for this finding, although it appears possible that BAP1 mutation may carry a higher risk of lethality in utero for males.

**Table 1:** Characteristics of the pooled families

**Family**	**Reference**	**Number of BAP1-mutated N=63**	**Number of BAP1-non-mutated N=55**
Family W	[6]	12	12
Family L	[6]	16	32
Family 1	[7]	4	3
Family 2	[7]	9	2
FUM036	[9]	8	1
Family 562	[10]	4	3
Family 729	[10]	10	2

**Table 2:** **Comparisons of gender, age and cancer rates between *BAP1*-mutated and non-mutated cohorts**

**Variable**	*BAP1*-mutated N=63*	***BAP1*-non-mutated N=55**	**p-value**	**OR (95%CI)**
Gender				
Male	22 (36.7%)	31 (56.4%)		
Female	38 (63.3%)	24 (43.6%)	0.034	-
Age of follow-up				
Number known	46	42		
Mean age (Std. Err.)	53.2 (2.1)	51.0 (2.3)	0.4851	-
At least 1 cancer				
Yes	40 (63.5%)	5 (9.1%)		17.39
No	23 (36.5%)	50 (90.9%)	<0.001	(6.07-49.83)
Malignant mesothelioma				
Yes	13 (21.0%)	0 (0.0%)		
No	49 (79.0%)	55 (100%)	<0.001	-
Uveal melanoma				
Yes	11 (17.7%)	0 (0.0%)		
No	51 (82.3%)	55 (100%)	0.001	-
Cutaneous melanoma				
Yes	8 (12.9%)	0 (0.0%)		
No	54 (87.1%)	55 (100%)	0.007^§^	-
MBAITs**				
Yes	24 (66.7%)	0 (0.0%)		
No	12 (33.3%)	12 (100%)	<0.001	-
Lung cancer				
Yes	3 (4.8%)	0 (0.0%)		
No	59 (95.2%)	55 (100%)	0.246^§^	-
Breast cancer***				
Yes	3 (7.9%)	1 (4.1%)		
No	35 (91.9%)	23 (95.9%)	0.621^§^	-
Renal cancer				
Yes	2 (3.3%)	0 (0.0%)		
No	60 (96.7%)	55 (100%)	0.497^§^	-

Abbreviations: OR: Odds Ratio; CI: confidence interval; *: 62 informative, 1 *BAP1*-mutated patient had cancer of unknown origin, **: calculated only in individuals belonging to present study, ref. 7 and ref. 10, ***: calculated only in women, -: not calculated; NS: non significant; Std. Err.: standard error; ^§^: two-tailed Fisher’s exact test.

The overall prevalence of cancer was significantly higher in the *BAP1*-mutated cohort compared with the non-mutated cohort (63.5% and 9.1% respectively, p < 0.001). Five tumors were observed in the non-mutated cohort: two prostate cancers, one breast cancer, one Hodgkin’s lymphoma and one Non-Hodgkin’s lymphoma. The odds ratio (OR) of cancer risk in the germline *BAP1*-mutated cohort versus the non-mutated cohort was 17.39 (95% CI: 6.07-49.83). MM, UVM, CM and MBAITs prevalence was significantly higher in the *BAP1*-mutated cohort compared with the non-mutated cohort (Table 2). No significant difference was found between the two cohorts concerning the rates of other cancers.

## Discussion

We performed a meta-analysis of all the published studies with *BAP1*-mutated families. To avoid the overestimation of the cancer risks estimates usually due to publication bias in meta-analyses, we chose to compare BAP1 mutated *vs* non-mutated patients from the same families. We demonstrate that germline *BAP1* mutations are associated with a significant increased overall risk of cancer and particularly of MM, UVM and CM (Table 2). In addition, two thirds of patients from the germline *BAP1*-mutated cohort presented MBAITs, while these tumors were not detected in the non-mutated individuals. Our results reveal that germline *BAP1* mutations cause a novel autosomal dominant hereditary cancer syndrome, the BAP1 cancer syndrome, characterized predominantly by MM, UVM, CM and by MBAITs and possibly by other cancers. In fact, because of the relatively high incidence of carcinomas in the general population, a larger number of *BAP1*-mutant family members have to be studied before ruling out the possibility that additional cancers are linked to the BAP1 cancer syndrome.

Early diagnosis is crucial for curative resection of CM and UVM. For its anatomical localization, MM is a tumor in which early diagnosis is particularly difficult; it is indeed often diagnosed in advanced stages when patients have median survivals of 6–12 months. However, when MM patients are diagnosed at Stage 1a, survivals of five or more years are not uncommon [11]. Indeed, a high degree of suspicion allowed us to detect four MM in the L and W families at an early stage and these patients experienced survivals of 5–10 and, hopefully, many more years.

MBAITs provide physicians with a marker to identify individuals who may carry germline *BAP1* mutations and thus are at high risk of developing CM, UVM and MM. We identified MBAITs among the majority of germline *BAP1* mutation carriers in the L and W families. Our meta-analysis demonstrates that the prevalence of MBAITs is significantly higher in germline *BAP1* mutation carriers compared to controls. MBAITs have variable papular macroscopic appearance similar to dermal nevi; nevertheless they present histological (Figure 2), immunohistochemical (Figure 2) and molecular (*BAP1* and *BRAF* mutations) features that allow their characterization in the broad spectrum of melanocytic lesions. We debated how to call these tumors, and we concluded that it was best to give them a new name to make a clear distinction between these tumors, and other melanocytic lesions, such as Spitz nevus and ASTs. Briefly, Spitz nevus consists of proliferation of large epithelioid or spindle shaped melanocytes, or a mixture of the two. At all ages, spindle cells are the most common cell type. Spitz nevi composed wholly of epithelioid cells occur mainly in early childhood. Spitz nevi go through the same junctional, compound and intradermal phases as common acquired nevi, but most are removed when they are compound lesions (when they have an epidermal and dermal component). Spitz nevi are roughly symmetrical and at any given level (epidermis, junction, upper dermis, lower dermis) the lesion shows similar architecture and cell type from side to side. The cellularity of the lesion and the size of the cells and their nuclei decrease toward the base of the Spitz nevus (so called maturation) and this is associated with loss of proliferative activity that is instead often present in the upper parts of the nevus. Also the architecture at the base (deep aspect) is infiltrative rather than expansile/pushing, the nevus cells lying dispersed between dermal collagen. In addition there is no nevus associate with them (unless it is part of a combined nevus). ATS are Spitzoid lesions that have some features that overlap with melanoma making the differential diagnosis challenging: for example, there is no maturation towards the deeper part of the dermis, and instead the AST cells show mitotic activity.

Instead, the tumors found in these BAP1 mutated patients show large epithelioid clonal cells (that resemble those found in Spitz nevus and in ASTs) but these cells are present only in the dermis (there is no epidermal component). In contrast to Spitz nevi there is no maturation towards the deeper part of the dermis, and in contrast to ASTs, Ki67 stain (a marker of cell proliferation) consistently showed absence of mitotic figures. In almost all of these BAP1 associated tumors, it was possible to detect remnants of a nearby conventional intradermal or compound nevus formed by smaller cells in close proximity to the large epithelioid cells, a very unlikely finding in Spitz and ASTs. In addition, at the cytophatological level, the nuclei of the large clonal cells in these BAP1 tumors are hyperchromatic, while in Spitz and ATS, the large cells have an open nuclear chromatin and a small distinct nucleolus. Also no Kamino bodies –common in Spitz- are detected in the BAP1 associated melanocytic tumors. At the molecular level, these lesions are characterized by BAP1 inactivation and almost always by concurrent BRAF mutation, features that are not found in Spitz and ASTs. Finally, in contrast to AST that at times mask a melanoma, these lesions are benign in appearance and behavior and had a history of being stable in morphology, per the patients, although it has been proposed that these tumors, that here we propose to name MBAITs, may rarely directly evolve into a CM, [7].

In summary, MBAITs have “Spitzoid features”, such as large epitheliod and spindle cells, pleomorphisms, etc., but are sufficiently distinct morphologically, cytologically, molecularly and clinically from ASTs that they should not be lumped together. The entity of AST has become a catch-all phrase to describe a very heterogenous group of lesions clinically and histologically. This has resulted in overly aggressive approaches to their management, such as the use of sentinel lymph node biopsy. Molecular genetic studies will ultimately help us divide the entity of ASTs into subgroups based on genetics and ultimately behavior. This progress will be valuable in guiding clinical management. Therefore, we believe that if we continue to lump different melanocytic lesions (such as MBAITs) under the umbrella term of AST we will miss an opportunity to help pathologists to identify and classify this subgroup and to guide clinicians in selecting patients who may need testing for BAP1 mutations.

The youngest individuals positively screened for MBAITs in the L and W families were in their third and fourth decades of life, but all of them described to us these lesions as having been present for several years. Accordingly, Wiesner *et al.* reported that in the *BAP1*-mutant families they studied, MBAITs appeared during the first two decades of life and increased in number with age [7,8]. Therefore, MBAITs may exhibit anticipation of several years to the development of MM, CM and UVM, the characteristic malignancies of the BAP1 cancer syndrome. Along the guidelines used for FAMM-P families [12] we advise families with hereditary *BAP1* mutation to have family members tested for mutant gene carrier status at the age of ten and, if positive, to begin routine screening with a total body dermatological examination as family members may develop melanoma at an early age. When these individuals become adults, annual eye examinations (indirect ophthalmoscopy, etc.) should be performed for early detection of UVM.

## Conclusions

Physicians should become aware that papular cutaneous tumors could be the first clinical manifestation of the BAP1 cancer syndrome. Suspicious lesions should be excised and evaluated by a pathologist. Pathologist also must become aware of the histological characteristics that characterize MBAITs from other intradermal melanocytic tumors. In the presence of intradermal tumors containing large epithelioid melanocytes, with marked cytologic atypia and pleomorphic, hyperchromatic nuclei, BAP1 immuno-staining should be performed. In the presence of negative nuclear BAP1 staining, the diagnosis of MBAIT is posed. Detection of *BRAF* mutations may help diagnose this condition in cases of inconclusive BAP1 staining. Next, genetic counseling should be offered and germline DNA should be tested for *BAP1* mutations to identify individuals and families at higher risk for one or more of the neoplasms associated with the BAP1 cancer syndrome. Germline BAP1 mutation carriers can be targeted for early detection and therapy that is associated with either a cure or improved prognosis.

## Abbreviations

AST: Atypical Spitz tumors; BAP1: BRCA1–associated protein 1; CM: Cutaneous melanoma; MBAITs: Melanocytic BAP1-mutated Atypical Intradermal Tumors; MM: Malignant mesothelioma; UVM: Uveal melanoma.

## Competing interests

MC has a patent pending on BAP1. MC has also received compensation for pathological diagnoses and honoraria for speaking engagements on the topics of genetics and mesothelioma. No other disclosures were reported. All authors read and approved the final manuscript.

## Authors’ contributions

MC designed the work, followed the L and W families over the past 12 years and conducted all of the field studies to collect specimens together with HY, studied the pathology with CAL, AP, TK, EH and wrote the manuscript with AN; LKF examined L and W family members for MBAITs, acquired the skin biopsies and contributed to the interpretation of the results and writing of the manuscript; FB conducted the meta-analysis and contributed to the writing of the manuscript; EGF collected data from family members and contributed to the writing of the manuscript; PBG conducted the molecular testing for BAP1 in a CLIA certified laboratory; GG contributed to the interpretation of the results and writing of the manuscript; RT facilitated the dermatological exams and helped acquire patient history in the L family; JF, TW, and HIP treated L and W family members over the years, contributed specimens and participated to the clinical evaluation and interpretation of the results. All authors read and approved the final manuscript.
